# Transcriptomic Changes Following Partial Depletion of CENP-E in Normal Human Fibroblasts

**DOI:** 10.3390/genes12091322

**Published:** 2021-08-26

**Authors:** Danilo Cilluffo, Roberta Flavia Chiavetta, Serena Bivona, Flavia Contino, Claudia Coronnello, Salvatore Feo, Aldo Di Leonardo, Viviana Barra

**Affiliations:** 1 Department of Biological, Chemical and Pharmaceutical Sciences and Technologies, University of Palermo, 90128 Palermo, Italy; cilluffo.danilo@gmail.com (D.C.); roberta.chiavetta@unipa.it (R.F.C.); serenabivona@gmail.com (S.B.); flaviacontino@hotmail.com (F.C.); salvatore.feo@unipa.it (S.F.); 2 Institute for Innovation and Biomedical Research (IRIB), CNR, 90146 Palermo, Italy; 3 Advanced Technology Network Center (ATEN), University of Palermo, 90128 Palermo, Italy; 4 Fondazione Ri.MED, 90133 Palermo, Italy; ccoronnello@fondazionerimed.com; 5 Centro di Oncobiologia Sperimentale (C.O.B.S.), Viale Delle Scienze, 90128 Palermo, Italy

**Keywords:** CENP-E, centromere, aneuploidy, cancer progression, expression profiling

## Abstract

The centromere is a fundamental chromosome structure in which the macro-molecular kinetochore assembles and is bound by spindle microtubules, allowing the segregation of sister chromatids during mitosis. Any alterations in kinetochore assembly or functioning or kinetochore–microtubule attachments jeopardize chromosome stability, leading to aneuploidy, a common feature of cancer cells. The spindle assembly checkpoint (SAC) supervises this process, ensuring a faithful segregation of chromosomes. CENP-E is both a protein of the kinetochore and a crucial component of the SAC required for kinetochore–microtubule capture and stable attachment, as well as congression of chromosomes to the metaphase plate. As the function of CENP-E is restricted to mitosis, its haploinsufficiency has been used to study the induced cell aneuploidy; however, the gene expression profile triggered by CENP-E reduction in normal cells has never been explored. To fill this gap, here we investigated whether a gene network exists that is associated with an siRNA-induced 50% reduction in CENP-E and consequent aneuploidy. Gene expression microarray analyses were performed at early and late timepoints after transfection. Initially, cell cycle regulation and stress response pathways were downregulated, while afterwards pathways involved in epithelial–mesenchymal transition, hypoxia and xenobiotic metabolism were altered. Collectively, our results suggest that CENP-E reduction triggers a gene expression program that recapitulates some features of tumor cells.

## 1. Introduction

The maintenance of chromosome segregation dynamics is essential to prevent numerical chromosome changes leading to aneuploidy. The centromere has an essential role in this process. Indeed, the centromere is the chromosome site where the multiproteic complex known as the kinetochore (kn) is built. This complex is required for the attachment of the microtubule fibres (mt) of the mitotic spindle to the chromosome, which is a necessary event for proper sister chromatid separation during anaphase. The spindle assembly checkpoint (SAC) in mitosis prevents the anaphase onset until all sister chromatids are properly attached by their kinetochores to the mitotic spindle [1,2]. A weakened SAC results in defective mitoses, which will generate aneuploidy, a common feature of cancer cells [3]. Centromere-associated protein-E (CENP-E) is an essential protein whose function is mainly played during mitosis. CENP-E is synthetized in the late S/G2 phase of the cell cycle, peaks in mitosis and is degraded in telophase [4,5]. CENP-E homozygous knockout (CENP-E^−/−^) mice have been shown to undergo massive chromosome segregation defects and death in embryonic stages. Nevertheless, heterozygous (CENP-E^+/−^) mice are viable even though they exhibit elevated levels of aneuploidy and develop spleen and lung cancers [6,7,8]. Previously, we also reported that CENP-E partial depletion in primary human fibroblasts (IMR90) and near diploid tumor cells (HCT116) is associated with aneuploidy, which is maintained up to two weeks [9]. On the other hand, upregulation of CENPE has been observed for some breast cancer cells, with a poor prognosis [10]; hence, CENPE could act both as an oncogene and as a tumor suppressor.

Firstly, CENP-E is a plus-end directed kinesin-7 motor bound to kinetochores, which has been identified as a component of the outer kinetochore [11] and has a role in the reinforcement of kn-mt binding, in the alignment to the spindle equator of mono-oriented chromosomes and in the spindle assembly checkpoint; however, how CENP-E is recruited in kinetochores is still debated. Recent works confirmed the previous observation that BubR1 is responsible for the initial recruitment of CENP-E in kinetochores [12], although other BubR1-indipendent mechanisms are involved in recruiting CENP-E in unattached kinetochores [13]. The role of CENP-E in ensuring stable kn-mt capture is finely regulated by aurora kinases A and B and PP1 with a phosphorylation–dephosphorylation switch mechanism [14]. It is well-known that mitosis is a target process used to harass cancer cells, and due to its critical role in accurate chromosome alignment, CENP-E might represent a promising target for several solid tumor therapies [15]. Given its highly specialized function in mitosis, alterations or reductions of CENP-E activity would affect specifically chromosome segregation, causing CIN and aneuploidy [7], although very little is known about the consequences of CENP-E activity reductions in terms of transcriptome deregulation and altered pathways or networks. In this regard, we previously showed that when aneuploidy is induced by silencing of different genes—specifically MAD2, pRb and DNMT1—in human primary fibroblasts, the cells undergo a common transcriptional change [16]. The altered expression of genes involved in cell cycle, specifically mitosis and G2-M transition, and in its regulation are mainly characterized by such transcriptional changes, suggesting the existence of a gene expression program typical of ‘early’ aneuploid cells.

Here, CENP-E was partially depleted by RNA interference in human primary fibroblasts (IMR90), while high-throughput gene expression profiling using DNA microarrays was performed at ‘early’ and ‘late’ timepoints to investigate which genes and networks are engaged by CENP-E reduction. Analysis of the transcriptional profiles at these timepoints revealed a number of downregulated genes associated with post-transcriptional silencing of CENP-E in aneuploid human primary fibroblasts. Gene set enrichment analysis showed different pathways that were significantly altered at both timepoints tested. Interestingly, at the early timepoint, we found downregulated pathways associated with cell cycle regulation (G2/M checkpoint and mitotic spindle) and the stress response (unfolded protein response and apoptosis); conversely, at the late timepoint, we found downregulated pathways correlated with tumor progression and metastasis (epithelial–mesenchymal transition, hypoxia and xenobiotic metabolism gene sets). These results will be helpful in better understanding how deregulation of crucial kinetochore-associated proteins influence genome-wide gene expression and pathway and network regulation in normal cells.

## 2. Materials and Methods

### 2.1. Cells and Culture Conditions

Primary human fibroblasts (IMR90, ATCC CCL-186™) were cultured in MEM medium supplemented with 10% fetal calf serum, 100 U/mL penicillin, 100 μg/mL streptomycin, 1 mM sodium pyruvate and 1% non-essential amino acids (all from Gibco, Life Technology, Monza, Italy). Cells were grown at 37 °C under 5% CO_2_ and atmospheric oxygen.

### 2.2. RNA Interference

For RNAi, cells were plated in 6-well dishes one day before transfection [17] and the siRNA transfection was performed as previously described [16]. Briefly, cells were transfected with a specific siRNA targeting CENP-E (siCENP-E: 5′-AAC GAAGAGUUACUUGGUGCCtt-3′) at a final concentration of 40 nM, which does not affect cell viability, as previously described [9]. An siRNA targeting the green fluorescent-protein (siGFP: 5′-GGCUACGUCCAGGAGCGCACCtt-3′) was used as the control. All siRNAs (21-nucleotide duplexes) were synthesized by Eurofins-Genomics.

### 2.3. Reverse Transcription Quantitative PCR (RT-qPCR)

Total RNA samples were extracted using a PureLink RNA Mini Kit (Ambion, Austin, TX, USA; Thermo Fisher Scientific, Waltham, MA, USA) and reverse transcription to cDNA was achieved by using the High-Capacity cDNA Reverse Transcription Kit (Applied Biosystems, Waltham, MA, USA; Thermo Fisher Scientific) according to the manufacture’s guidelines. Gene expression analysis was performed via RT-qPCR, as previously described [16], with specific forward and reverse primers: GAPDH (Fw: 5′-CTCATGACCACAGTCCATGCC-3′, Rev: 5′-GCCATCCACAGTCTTCTGGGT-3′), CENP-E (Fw: 5′-GTGGGACCAGTTCAGCCTGATA-3′, Rev: 5′-GATGTGAACCACGAAAACCCTC-3′), DUSP6 (Fw: 5′-AACAGGGTTCCAGCACAGCAG-3′, Rev: 5′-GGCCAGACACATTCCAGCAA-3′) BTG2 (Fw: 5′-CTCCATCTGCGTCTTGTACGA-3′, Rev.: 5′-AGACTGCCATCACGTAGTTCT-3′), CYFIP2 (Fw: 5′-TCCGTATCCACCGTCCAAT-3′, Rev.: 5′- AATCTCCAGCAGCCACTCC-3′) KLF4 (Fw: 5′-GCAATATAAGCATAAAAGATCACC-3′, Rev: 5′- AACCAAGACTCACCAAGCACC-3′) TFPI2 (Fw: 5′-AACGCCAACAATTTCTACACCT-3′, Rev: 5′- TACTTTTCTGTGGACCCCTCAC-3′). The fold change of each gene was normalized to the housekeeping gene GAPDH.

### 2.4. Microarray and Bioinformatics Analysis

The gene expression microarrays were performed as previously described [16]. Extracted RNA samples were analyzed for quantity and quality using NanoDrop ND-1000 (Thermo Fisher Scientific) and an Agilent 2100 bioanalyzer (minimum RIN score of 8). Analyzed samples included three clusters with three independent biological replicates (siCENP-E 72 h, siCENP-E 2 w and siGFP control cells). Samples labelling, hybridization on separate Whole Human Genome Microarray 4 × 44 K (Agilent-G4112F) arrays and image analysis were performed as described in [16]. Statistical data analysis and normalization were conducted with R software, with the threshold set as log (fold change) >1.4 to select differentially expressed genes (DEGs) in the 72 h and 2 w samples with respect to control (siGFP). Statistically significant differences were inferred from the Student’s *t*-test with a *p*-value cut-off of 0.05. Unsupervised hierarchical clustering was performed using the Euclidean distance and the average linkage method. FunRich software [18] was used to cluster DEGs shared by the two samples and lists of Gene Ontology (GO) terms (Cellular Component, Molecular Function, Biological Process), as well as the specific biological pathways to which these gene belong.

### 2.5. Gene Set Enrichment Analysis (GSEA)

Microrray data for the two samples of the siRNA-treated (72 h and 2 w) IMR90 cells were also analyzed using the Gene Set Enrichment Analysis (GSEA) from the Broad Institute tool in terms of altered gene sets [19,20]. The ES distribution was generated using 1000 gene permutations, then a normalization enrichment score (NES) was associated with each pathway. Hallmark collection was used as the gene set database. Gene sets with *p*-values < 0.05 were considered statistically significant.

## 3. Results

### 3.1. Transcriptome and Bioinformatics Analyses

We mimicked CENP-E haploinsufficiency in IMR90 cells using RNA interference (hereafter named IMR90-siCENP-E cells) [9] and then analyzed the transcriptome at early (seventy-two hours, 72 h) and late (two weeks, 2 w) timepoints after siRNAs transfection. To this end, we used a gene expression microarray and compared IMR90 siCENP-E cells with control cells (IMR90-siGFP) to find a specific gene expression profile triggered by CENP-E reduction in normal human fibroblasts.

The dendrogram in Figure S1 shows the hierarchical clustering between IMR90 control cells and siCENP-E at 72 h and 2 w.

To detect the differentially expressed genes (DEGs), data from three replicated experiments were arranged and each expression profile was annotated, filtered and ranked to provide the final gene list. DEGs with a significance level of *p* < 0.05 were chosen, while to highlight slight differences in the expression levels we also selected DEGs with a fold change >1.4.

**Figure 1:**
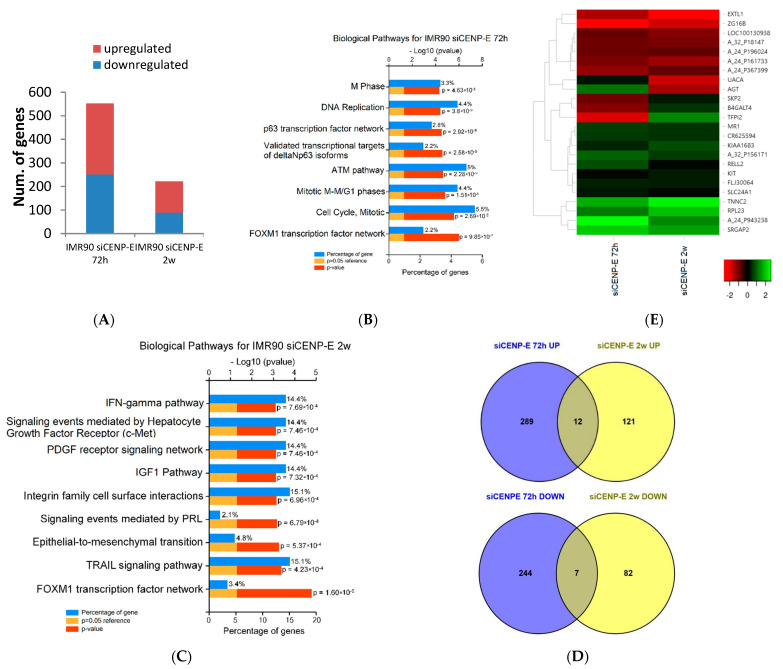
Genome-wide microarray analysis. Comprehensive gene expression analysis at early (72 h) and late (2 w) timepoints after siRNAs (siCENP-E) transfection of IMR90 cells compared to IMR90 control cells (siGFP). (**A**) Histogram showing number of genes up or down regulated in each sample. (FC ± 1.4, *p*-value cutoff ≤ 0.05) (**B**,**C**) Biological pathways for DEGs. *x*-axis represents the percentages of genes or −log10 values (*p*-value); *y*-axis represents the Biological pathways significantly enriched using the FunRich bioinformatics tool. (**D**) Venn diagram set out the number of common up- (**top**) and down- (**bottom**) regulated DEGs found in the two samples. (**E**) Heat map displaying expression levels of significantly common DEGs in trasfected IMR90 cells.

This analysis highlighted 552 DEGs in IMR90-siCENP-E 72 h (301 upregulated, 251 downregulated) and 222 DEGs in IMR90-siCENP-E 2 w (133 upregulated, 89 downregulated) (Figure 1A). Among the DEGs, we observed significant downregulation of the targeted gene CENP-E at 72 h post-transfection (logFC-1.26, *p*-value 0.009), confirming the functionality of the used siRNAs, our previous data [9] and the reliability of our microarray analysis. On the other hand, at the late timepoint, the CENP-E transcript value was not significantly downregulated, suggesting that cells were not exposed anymore to siRNAs targeting CENP-E.

A Funrich V3 tool was used to perform a Gene Ontology (GO) analysis of the listed DEGs to characterize the early and late effects of CENP-E downregulation on cellular physiology. At 72 h from silencing, 11 molecular function (MF) terms were significantly enriched, including metallopeptidase activity, protein serine–threonine kinase, phosphatase activities and RNA-directed DNA polymerase activity. In addition, 19 cellular component (CC) terms were significantly enriched, including spindle microtubule, nucleoplasm and chromosome centromeric region. Moreover, 22 terms were significantly enriched among the biological process terms, including regulation of cell cycle, lipid metabolism and cell growth or maintenance (Table S1).

At 2 weeks from CENP-E silencing, 9 molecular function (MF) terms were significantly enriched, including phosphoric diester hydrolase activity and MHC classes I and II receptor activity. Seven cellular component (CC) terms were significantly enriched, including extracellular space and SCF ubiquitin ligase complex. In addition, 6 terms were significantly enriched among the biological process terms, including carbohydrate-mediated signaling, lipid metabolism and signal transduction (Table S2).

We also clustered significantly DEGs depending on their role in specific biological pathways. This analysis revealed a set of genes belonging to the FOXM1 transcription factor network, mitotic pathway, cell cycle, mitotic M-M/G1 phase and ATM pathway at an early timepoint. On the other hand, biological pathways characterizing the late timepoint included the TRAIL signaling pathway, epithelial-to-mesenchymal transition and integrin family cell surface interactions (Figure 1B,C). More details can be found in Tables S3 and S4.

Moreover, we identified DEGs shared by IMR90 cells after CENP-E partial depletion at both timepoints (Table 1).

Nineteen DEGs (12 upregulated and 7 downregulated) expressed in a coordinated manner (upregulated or downregulated in both samples), were shared between the two samples (Figure 1D,E).

### 3.2. Validation of Arrays Data Using RT-qPCR Analysis

**Figure 2:**
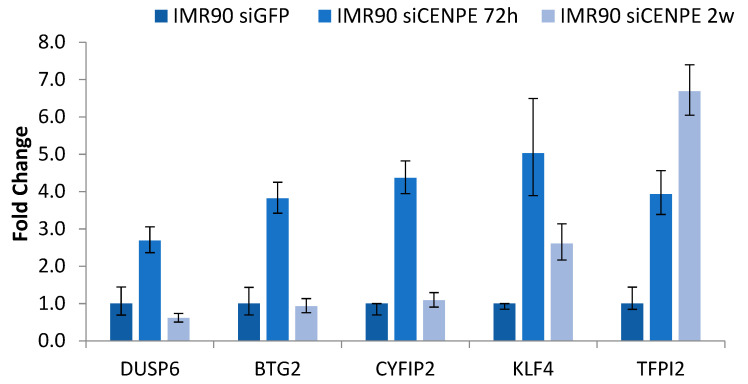
Relative mRNA expression levels of specific downregulated genes. RT-qPCR results from siCENP-E 72 h and siCENP-E 2 w RNA-interfered IMR90 cells compared to siGFP-transfected cells. The analysis were performed in triplicate. The data presented are the means ± SD (error bar) of fold changes.

We validated the observed gene expression changes found with the microarray analysis via RT-qPCR in independent biological replicates of IMR90 cells treated with the CENP-E siRNA under the same conditions used for the microarrays analysis. Five DEGs were randomly selected for RT-qPCR: DUSP6, BTG2, CYFIP2, KLF4 and TFPI2 (Figure 2). Gene fold changes resulting from RT-qPCR were in agreement with the microarray data. Although there was a small difference in the fold change values between the two methods, generally the results were correlated.

### 3.3. Gene Set Enrichment Analysis (GSEA)

Microarray data were further analyzed with the GSEA bioinformatics software [21] to identify gene sets significantly downregulated at early (72 h) and late (2 w) timepoints in IMR90-siCENP-E cells with respect to control cells (siGFP). This analysis revealed a pattern of pathways and networks being altered in transfected human primary fibroblasts at the two timepoints. Table 2 shows the significant (FDR *q*-val < 0.2) downregulated gene sets. Among the gene sets significantly enriched in IMR90-siCENP-E 72 h, two were found to be upregulated, namely cholesterol homeostasis and apoptosis, while ten were found to be downregulated, including G2/M checkpoint and mitotic spindle gene sets. On the other hand, GSEA analysis in IMR90-siCENP-E 2 w cells showed fifteen gene sets to be significantly enriched, eleven of which were upregulated, including epithelial–mesenchymal transition, interferon gamma response and hypoxia, with four downregulated gene sets, including mTORC1 signalling and P53 pathway.

**Figure 3:**
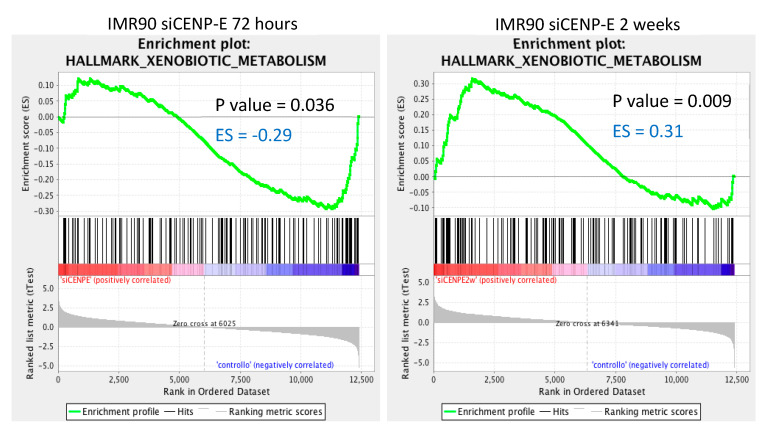
Enrichment plots of the significantly enriched xenobiotic metabolism gene set shared by the two samples. The *y*-axis show the enrichment scores (ES) and the *x*-axis shows gene members for the two gene sets. The green line connects points between ES and genes. ES represent the amount to which a gene is over-represented in a gene set. The coloured bands represent positive (red) or negative (blue) degrees of correlation of genes with the siCENP-E (72 h or 2 w) phenotype. The xenobiotic metabolism gene set was negatively correlated (ES = −0.29) with the early siCENP-E timepoint; 26 of 145 genes from this set were downregulated at 72 h with respect to the control. Conversely, the xenobiotic metabolism gene set was positively correlated (ES = 0.31) with the late siCENP-E timepoint and 34 of 145 genes from this set were upregulated at 2 weeks with respect to the control (Tables S5 and S6). Significance threshold set at *p*-value < 0.05. The enrichment plots were derived from GSEA tool [21].

Interestingly, we found that only one gene set was shared but differently downregulated in both early and late siCENP-E IMR90 cells—the xenobiotic metabolism hallmark (Figure 3). The expression levels of five genes belonging to this gene set (TPST1, ATOH8, MAOA, TYR, AKR1C2) were altered in the two samples (72 h and 2 w), although in opposite directions, being downregulated in early siCENP-E cells and upregulated in late siCENP-E cells (Tables S5 and S6).

Deepening the analysis of this specific downregulated gene set, we focused on the mitotic spindle gene set, which was significantly enriched in the IMR90-siCENP-E 72 h sample. We found that forty-two genes belonging to this gene set were downregulated (Table 3). Among these, eight are mitotic kinesins and spindle-associated genes (CENP-F, CENP-J, KIF2C, KIF4A, KIF23, KIF15, CKAP5, TPX2). In particular, CENP-F and CENP-J are centromere proteins associated with kinetochores, similarly to CENP-E.

Moreover, the study of the DEG members from each gene set downregulated in the two samples revealed a list of genes altered at both timepoints, although belonging to different pathways. We found two genes (BTG2 and DDIT3) upregulated at 72 h from CENP-E knockdown and downregulated at 2 w belonging to apoptosis and P53 pathway gene sets and shared among the two samples.

## 4. Discussion

Kinesin-like protein CENP-E is a critical component of the kinetochore that assures correct chromosome biorientation during mitosis. As a result, it is well known that partial loss or drug inhibition of CENP-E causes mitotic errors and aneuploidy in human cells [6,9,22]; however, the effects of CENP-E downregulation on gene expression are largely unknown. In this study, we comprehensively characterize for the first time the effects of CENP-E transient partial knockdown on transcriptomics in normal human cells.

Our previous studies showed that CENP-E downregulation induced aneuploidy in human fibroblasts and in near diploid cancer cells, which is maintained for at least two weeks [9]. In the current study, we expand on these findings to unveil the transcriptional program changes that cells undertake in response to the stressful condition correlated with CENP-E partial depletion.

We mimicked CENP-E haploinsufficiency in primary human fibroblasts IMR90 via RNA interference and followed these cells up to 2 weeks from siRNA transfection. The whole transcriptome was analyzed by microarray at 72 h (early) and 2 w (late) from siRNA transfection to evaluate the short- and long-term transcriptional consequences.

We performed a detailed analysis of the transcriptional changes occurring in human primary fibroblasts via DNA microarray. Using bioinformatics tools (R, FunRich and GSEA), we listed several differentially expressed genes to identify a list of common expressed genes, pathways and regulatory networks.

We found 552 downregulated genes at the early timepoint, showing that although CENP-E functions mainly during chromosome segregation, its downregulation also has pleiotropic effects involving several kinds of genes. Moreover, at the “late” timepoint, the number of differentially expressed genes was strongly reduced (222), which was most probably due to the ability of normal primary cells to progressively restore themselves to a steady state once the alteration (CENP-E reduction) exhausted itself; however, two weeks after siCENP-E transfection, there was still a non-negligible number of differentially expressed genes, which suggests that downregulation of CENP-E also causes long-term effects in cell homeostasis. Considering that until now CENP-E has never been found to be directly or indirectly involved in gene transcription (by binding transcription factors for example), we can infer that the observed transcriptional change is triggered by the cell condition that is established upon the reduction of CENP-E. We suggest that two cell conditions are induced by CENP-E reduction: (i) alteration of mitosis; (ii) aneuploidy. Since we looked at cells at 72 h after siRNA transfection, and even after 2 weeks, several or many generations from the first altered mitosis, and based to our previous work [16] (as discussed later), we strongly suggest that aneuploidy, in fact, triggered the transcriptional changes.

We found gene sets that were significantly associated with CENP-E-downregulated cells in respect to the control. GSEA analysis showed that ‘E2F targets’, ‘mitotic Spindle’ and ‘G2/M checkpoint’ pathways were downregulated at the ‘early’ timepoint, suggesting that CENP-E reduction rapidly weakens the mechanisms controlling cell cycle progression. This is in accordance with the ‘early aneuploidy gene expression signature’ we previously observed in aneuploid IMR90 cells [16]. In this work, we showed that fibroblasts, when aneuploidy was induced by the single downregulation of three different genes (MAD2, pRb and DNMT1), underwent a common transcriptional reprogramming process. The GSEA analysis highlighted that ‘mitotic spindle’, ‘E2F target’ and ‘G2/M checkpoint’ gene sets were downregulated similarly to human primary fibroblasts at the early timepoint after RNA interference targeting CENP-E and not at the late timepoint (discussed later). This confirms the existence of an early aneuploidy gene expression signature. It is noteworthy that CENP-E reduction affects the gene expression of other centromere proteins, such as CENP-J and CENP-F. Intriguingly, CENP-F and CENP-E are considered paralogs and were recently found to specifically interact with BUB1 and BUBR1, respectively, which are themselves paralogs acting in the SAC [23]. In addition, we also found alterations in the ‘unfolded protein response’ pathway, dysfunctions of which have been linked to genomic instability [24].

The analysis of the transcriptome of IMR90 siCENP-E cells at the late timepoint did not highlight changes in the expression of genes involved in the cell cycle progression and checkpoints, suggesting that the disorder in such pathways is only a first response to the reduction of the mitotic protein CENP-E. Once the siRNAs targeting CENP-E are exhausted and CENP-E transcripts are restored, cell cycle can be considered safe and the related gene expression comes back to the control condition; however, other gene sets appeared to be altered at the late timepoint. Among these gene sets, it is worth mentioning the epithelial–mesenchymal transition, which is largely known to be crucial for malignant progression [25]. This observation could imply that an insult to a centromere protein, even if solved, quickly opens the door to cell transformation. Similarly, at the late timepoint, CENP-E downregulation triggers the upregulation of the hypoxia gene set, predisposing cells to adaptation to a low-oxygen environment, which notoriously influences tumoral mass. Additionally, at the late timepoint, we detected transcriptional changes in cell metabolisms (xenobiotic and heme metabolisms), highlighting the metabolic switch typical of cancer cells. Gene sets involved in the inflammatory response are also upregulated at the late timepoint after siCENP-E transfection.

## 5. Conclusions

Collectively, our results show that the reduction of the centromere protein CENP-E, which is known to function only in mitosis, triggers a transcriptional program change that initially involves mainly cell cycle genes and afterwards includes a plethora of genes playing roles in pathways that are also downregulated in cancer cells.

## Figures and Tables

**Table 1:** List of common deregulated genes shared by IMR90 siCENP-E at 72 h and 2 weeks with relative Log (Fold Change) values (only annotated probes are reported).

*Gene ID*	Description	LogFC		# Chr.	Map Location
		72 h	2 Weeks		
ZG16B	zymogen granule protein 16B	−2.21378	−1.58077	16	16p13.3
TFPI2	tissue factor pathway inhibitor 2	−1.73241	1.967329	7	7q22
EXTL1	Exostosin-like glycosyltransferase 1	−1.27449	−2.37647	1	1p36.1
MR1	major histocompatibility complex, class I-related	1.173685	1.064675	1	1q25.3
AGT	angiotensinogen (serpin peptidase inhibitor, clade A, member 8)	1.752677	−1.15911	1	1q42.2
RPL23	ribosomal protein L23	1.819593	2.630793	17	17q
TNNC2	troponin C type 2 (fast)	2.405029	3.232683	20	20q12-q13.11
SRGAP2	SLIT-ROBO Rho GTPase activating protein 2	2.733915	2.305187	1	1q32.1
B4GALT4	UDP-Gal:betaGlcNAc beta 1,4- galactosyltransferase, polypeptide 4	−0.88068	1.067247	3	3q13.3
UACA	uveal autoantigen with coiled-coil domains and ankyrin repeats	0.669221	−1.5223	15	15q22-q24
KIAA1683	Q Domain-Containing Protein N	0.875501	1.339538	19	19p13.1
SKP2	S-phase kinase−associated protein 2, E3 ubiquitin protein ligase	−0.68236	0.753601	5	5p13
KIT	v-kit Hardy-Zuckerman 4 feline sarcoma viral oncogene homolog	0.552419	0.785465	4	4q12
RELL2	RELT-like 2	1.337275	0.531247	5	5q31.3
SLC24A1	solute carrier family 24 (sodium/potassium/calcium exchanger), member 1	0.701308	0.546544	15	15q22

**Table 2:** Gene sets enriched in CENP-E transient knocked-down IMR90 cells.

*Hallmark Gene set 72 h*	*SIZE*	*ES*	*NES*	*p-Value*	*FDR q-Value*
*Upregulated*					
Cholesterol Homeostasis	55	0.550825	2.219.529	0.0	0.0
Apoptosis	128	0.29306307	14.145.108	0.026666667	0.17741449
*Downregulated*					
E2F Targets	124	−0.4112242	−18.818.628	0	0.001760578
G2/M Checkpoint	130	−0.42952272	−19.638.364	0	0.001968572
Mitotic Spindle	132	−0.33654705	−15.422.429	0	0.08193885
Angiogenesis	25	−0.44820115	−14.857.963	0.04587156	0.100587666
Estrogen Response Late	160	−0.30400178	−14.472.796	0.013745705	0.11437272
Bile Acid Metabolism	81	−0.33262056	−14.141.632	0.028520498	0.12994246
Xenobiotic Metabolism	145	−0.29437807	−13.890.945	0.036036037	0.14013477
Unfolded Protein Response	74	−0.31752646	−13.405.416	0.060998153	0.14493392
Adipogenesis	130	−0.29467914	−1.363.687	0.03345725	0.15041217
TNFA Signaling via NFkB	142	−0.28777105	−13.413.501	0.041736227	0.15983883
***Hallmark Gene set* 2 weeks **	*SIZE*	*ES*	*NES*	*p-vauel*	*FDR q-value*
*Upregulated*					
Epithelial Mesenchymal Transition	151	0.33510843	1.519.574	0.0075642965	0.11385157
IL6/JAK/STAT Signaling	58	0.37629685	14.682.375	0.034035657	0.11548446
Interferone Gamma Response	129	0.33727473	14.752.915	0.012403101	0.1355435
Hypoxia	141	0.34279573	15.343.299	0.00147929	0.14622506
Xenobiotic Metabolism	145	0.31554386	14.156.777	0.009036144	0.15259206
Coagulation	100	0.32489774	13.754.646	0.032357473	0.16717246
Heme Metabolism	136	0.30528104	13.594.577	0.03153153	0.16950405
Complement	141	0.31029537	13.893.101	0.02413273	0.16991296
Hedgeog Signaling	24	0.41114244	13.165.202	0.12477396	0.17681527
Inflammatory Response	138	0.30268276	13.413.032	0.052469134	0.17737256
UV Response DN	104	0.3095823	13.233.161	0.046178345	0.18309012
*Downregulated*					
mTORC1 Signaling	141	−0.33381698	−16.185.987	0.0	0.047431484
P53 Pathway	135	−0.34690675	−16.548.364	0.005830904	0.06875795
NOTCH Signaling	24	−0.45094362	−14.955.359	0.04481132	0.09119969
KRAS Signaling UP	143	−0.29774994	−14.312.139	0.018018018	0.1097472

ES: Enrichment Score; NES: Normalized Enrichment Score; FDR: False Discovery Rate.

**Table 3:** Hallmark mitotic-spindle-enriched genes in IMR90 siCENP-E 72 h cells.

Gene Symbol	Description	Running ES
TIAM1	T-cell lymphoma invasion and metastasis 1	−0.32938716
LRPPRC	Leucine-rich PPR-motif-containing	−0.32254452
FSCN1	fascin homolog 1, actin-bundling protein (Strongylocentrotus purpuratus)	−0.31544223
HDAC6	histone deacetylase 6	−0.31205016
UXT	Ubiquitously-expressed transcript	−0.3070534
TUBD1	tubulin, delta 1	−0.30694872
CKAP5	Cytoskeleton-associated protein 5	−0.3005278
TTK	TTK protein kinase	−0.2928824
ARHGAP4	Rho GTPase-activating protein 4	−0.29161006
MARCKS	myristoylated alanine-rich protein kinase C substrate	−0.29939783
GEMIN4	gem (nuclear organelle)-associated protein 4	−0.29208082
FBXO5	F-box protein 5	−0.2945973
ROCK1	Rho-associated, coiled-coil-containing protein kinase 1	−0.29402748
LLGL1	lethal giant larvae homolog 1 (Drosophila)	−0.29998654
PXN	Paxillin	−0.30580252
CENPF	centromere protein F, 350/400 ka (mitosin)	−0.29683083
PREX1	Phosphatidylinositol 3,4,5-Trisphosphate-Dependent Rac Exchanger 1 Protein	−0.29543936
RAB3GAP1	RAB3 GTPase-activating protein subunit 1 (catalytic)	−0.28851372
CENPJ	centromere protein J	−0.28630605
KIF2C	kinesin family member 2C	−0.27653277
TPX2	TPX2, microtubule-associated, homolog (Xenopus laevis)	−0.26817042
TSC1	tuberous sclerosis 1	−0.2579142
NCK1	NCK adaptor protein 1	−0.25523746
ARHGEF3	Rho guanine nucleotide exchange factor (GEF) 3	−0.2437383
SYNPO	Synaptopodin	−0.2319448
STK38L	Serine–threonine kinase 38-like	−0.22208665
OPHN1	oligophrenin 1	−0.21298712
BUB1	BUB1 budding uninhibited by benzimidazoles 1 homolog (yeast)	−0.20986164
KIF4A	kinesin family member 4°	−0.20093325
ARHGAP29	Rho GTPase-activating protein 29	−0.18930057
PRC1	protein regulator of cytokinesis 1	−0.17876585
CLASP1	Cytoplasmic-linker-associated protein 1	−0.1655671
TOP2A	topoisomerase (DNA) II alpha 170kDa	−0.16242237
RHOF	ras homolog gene family, member F (in filopodia)	−0.14529541
KIF15	kinesin family member 15	−0.12773909
PCGF5	polycomb group ring finger 5	−0.11064719
NEK2	NIMA (never in mitosis gene a)-related kinase 2	−0.09230707
CCNB2	cyclin B2	−0.076223895
KIF23	kinesin family member 23	−0.058513902
RACGAP1	Rac GTPase-activating protein 1	−0.03790126
CENPE	centromere protein E, 312 kDa	−0.018124685
KIF11	kinesin family member 11	0.005052448

## Data Availability

Microarray data have been deposited into NCBI’s Gene Expression Omnibus (GEO) database and can be accessed via accession number GSE181191.

## Supplementary Materials

The following are available online at www.mdpi.com/2073-4425/12/9/1322/s1. Supplementary Figure S1 shows the unsupervised hierarchical clustering dendrogram comparing IMR90 cells transfected with siGFP (control) and siRNA targeting CENP-E at 72 h and 2 weeks. Supplementary Table S1 indicates the Gene Ontology (GO) terms (cellular component, molecular function and biological processes) enriched in the IMR90 siCENP-E 72 h sample (*p*-value < 0.05. Supplementary Table S2 indicates the Gene Ontology (GO) terms (cellular component, molecular function and biological processes) enriched in the IMR90 siCENP-E 2 w sample (*p*-value < 0.05). Supplementary Table S3 lists the top 20 biological pathways enriched in the IMR90 siCENP-E 72 h sample (*p*-value < 0.01). Supplementary Table S4 lists the top 20 biological pathways enriched in the IMR90 siCENP-E 2 w sample (*p*-value < 0.01). Supplementary Table S5 indicates the xenobiotic-metabolism-enriched genes in the IMR90 siCENP-E 72 h sample. Supplementary Table S6 indicates the xenobiotic-metabolism-enriched genes in the IMR90 siCENP-E 2 w sample.
