# Supplementary figures and images for: Transcriptomic Changes Following Partial Depletion of CENP-E in Normal Human Fibroblasts
Source: Genes (Basel). 2021 Aug 26;12(9):1322. doi: 10.3390/genes12091322 (PMC8466516; doi:10.3390/genes12091322)

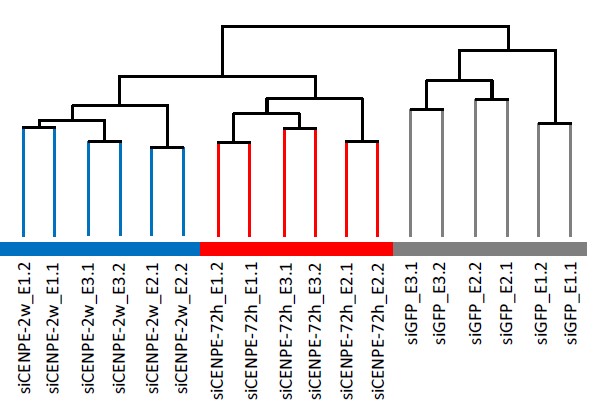

Supplement: Supplementary file 1 [file genes-12-01322-s001.zip › Supplementary Figure S1.jpg]
